# CCR4-Not Complex Subunit Not2 Plays Critical Roles in Vegetative Growth, Conidiation and Virulence in Watermelon Fusarium Wilt Pathogen *Fusarium oxysporum* f. sp. *niveum*

**DOI:** 10.3389/fmicb.2016.01449

**Published:** 2016-09-16

**Authors:** Yi Dai, Zhongye Cao, Lihong Huang, Shixia Liu, Zhihui Shen, Yuyan Wang, Hui Wang, Huijuan Zhang, Dayong Li, Fengming Song

**Affiliations:** State Key Laboratory for Rice Biology, Institute of Biotechnology, Zhejiang UniversityHangzhou, China

**Keywords:** watermelon (*Citrullus lanatus*), *Fusarium oxysporum* f. sp. *niveum*, CCR4-Not complex, Not2, virulence, conidiogenesis, cell wall integrity, oxidative response

## Abstract

CCR4-Not complex is a multifunctional regulator that plays important roles in multiple cellular processes in eukaryotes. In the present study, the biological function of FonNot2, a core subunit of the CCR4-Not complex, was explored in *Fusarium oxysporum* f. sp. *niveum* (*Fon*), the causal agent of watermelon wilt disease. *FonNot2* was expressed at higher levels in conidia and germinating conidia and during infection in *Fon*-inoculated watermelon roots than in mycelia. Targeted disruption of *FonNot2* resulted in retarded vegetative growth, reduced conidia production, abnormal conidial morphology, and reduced virulence on watermelon. Scanning electron microscopy observation of infection behaviors and qRT-PCR analysis of *in planta* fungal growth revealed that the Δ*FonNot2* mutant was defective in the ability to penetrate watermelon roots and showed reduced fungal biomass in root and stem of the inoculated plants. Phenotypic and biochemical analyses indicated that the Δ*FonNot2* mutant displayed hypersensitivity to cell wall perturbing agents (e.g., Congo Red and Calcofluor White) and oxidative stress (e.g., H_2_O_2_ and paraquat), decreased fusaric acid content, and reduced reactive oxygen species (ROS) production during spore germination. Our data demonstrate that *FonNot2* plays critical roles in regulating vegetable growth, conidiogenesis and conidia morphology, and virulence on watermelon via modulating cell wall integrity, oxidative stress response, ROS production and FA biosynthesis through the regulation of transcription of genes involved in multiple pathways.

## Introduction

The soil-borne ascomycete *Fusarium oxysporum* is a species complex with more than 80 plant host-specific *formae speciales* (Kistler et al., 1998; Michielse and Rep, 2009). *F. oxysporum* is an important fungal pathogen and causes vascular wilt disease on over 100 different plant species leading to significant crop losses worldwide. The infection process of *F. oxysporum* toward roots of host plants includes several steps: root recognition, root surface attachment and colonization, penetration and colonization of the root cortex and, hyphal proliferation within the xylem vessels (Michielse and Rep, 2009). Hyphae generated from germinated spores first colonize the root surface, directly penetrate the root epidermal layer (Pérez-Nadales and Di Pietro, 2011), advance inter- and intracellularly through the cortex and finally use the xylem vessels as avenues to enter and colonize the stem of host plants (Lagopodi et al., 2002).

During the last two decades, interactions of *F. oxysporum* with tomato and *Arabidopsis* plants have been developed as model systems, with which extensive studies have greatly advanced our understanding on the molecular mechanisms of pathogenicity and host infection of *F. oxysporum*. A quite number of pathogenicity genes in *F. oxysporum* have been identified through application of genetic approaches such as random insertional mutagenesis and targeted disruption of genes of interest (Michielse and Rep, 2009; Michielse et al., 2009a; Ma et al., 2010; Ma L. J.et al., 2013). These identified pathogenicity genes have been shown to play roles in modulation of directed hyphal growth, root penetration and invasion growth during different stages of pathogenesis. Once spores germinated in the soil, hyphae grow toward the roots of host plants in a chemotropism-directed manner and this chemotropism-directed hyphal growth in *F. oxysporum* is mediated by distinct MAPK modules of FMK1 and MPK1 for nutrients and sex pheromones, respectively (Turrà et al., 2015). During interaction with host plants, *F. oxysporum* secretes a large number of small proteins including the secreted in xylem proteins (Houterman et al., 2007; Ma et al., 2010; Takken and Rep, 2010; Schmidt et al., 2013; de Sain and Rep, 2015; Gawehns et al., 2015), which act as virulence factors (Thatcher et al., 2012; Gawehns et al., 2014) or modulators of plant immune response (Rep et al., 2004; Houterman et al., 2008, 2009; Ma L.et al., 2013; Ma et al., 2015). Many fungal genes have been shown to be essential for full virulence of *F. oxysporum* including G-protein subunits (Jain et al., 2002, 2003) and Rho-type GTPase Rho1 (Martínez-Rocha et al., 2008), several protein kinases such as MAP kinases (Di Pietro et al., 2001; Ding et al., 2015; Turrà et al., 2015), two-component histidine kinase (Rispail and Di Pietro, 2010) and cAMP-dependent protein kinase A (Kim et al., 2011), transcription factors such as REN1, FOW2, SGE1, FTF1, PacC, Ctf1, xlnR, Snt2, fost12, HpaX and Con7-1 (Caracuel et al., 2003; Ohara et al., 2004; Imazaki et al., 2007; Ramos et al., 2007; Calero-Nieto et al., 2007; Rocha et al., 2008; Michielse et al., 2009b; Rispail and Di Pietro, 2009; Asunción García-Sánchez et al., 2010; Denisov et al., 2011; López-Berges et al., 2012; Ruiz-Roldán et al., 2015; Niño-Sánchez et al., 2016), F-box protein FRP1 and its interactor CRE (Duyvesteijn et al., 2005; Jonkers and Rep, 2009; Jonkers et al., 2011), velvet complex (López-Berges et al., 2013), membrane protein Msb2 and Sho1 (Pérez-Nadales and Di Pietro, 2011, 2015), mitochondrial protein FOW1 (Inoue et al., 2002) and co-chaperone Dnj1 (Lo Presti et al., 2016). In addition, genes encoding for chitin synthases (Madrid et al., 2003; Martín-Urdíroz et al., 2008; Martín-Udíroz et al., 2004), alcohol dehydrogenase (Corrales Escobosa et al., 2011), pisatin demethylase (Coleman et al., 2011), tomatinase (Pareja-Jaime et al., 2008), glucanosyltransferase (Caracuel et al., 2005), *N*-acetylglucosamine transferase (López-Fernández et al., 2013), mannosyltransferase (Li et al., 2014), 3-carboxy-*cis, cis*-muconate lactonizing enzyme (Michielse et al., 2012), and other enzymes involved in arginine biosynthesis (Namiki et al., 2001), glycogen catabolism (Corral-Ramos and Roncero, 2015) were also shown to play roles in virulence of *F. oxysporum*. Thus, it is clear that *F. oxysporum* pathogenicity is regulated by a complicated network, which includes signal perception, transduction, gene transcription, and metabolism.

The CARBON CATABOLITE REPRESSION4 (CCR4)-Negative on TATAless (Not) complex is a large (>2 MDa) multi-subunit, multi-functional eukaryotic regulator that serves as a control node for integration of environmental signals into cellular physiology as well as acts as a coordinator of multiple nuclear and cytoplasmic steps in gene expression (Collart and Panasenko, 2012; Panepinto et al., 2013; Inada and Makino, 2014). The CCR4-Not complex is composed of nine core subunits, which play roles in gene transcription, posttranscriptional regulation of mRNA stability, export of mRNAs from the nucleus to the cytoplasm and quality control (Panepinto et al., 2013). Among the subunits, Not2 is one of the core members in the CCR4-Not complex and is an evolutionarily conserved protein in eukaryotes (Anand et al., 2007). Recently, Not2 was found to bind RNA Pol II directly and promote transcriptional elongation, revealing the fundamental involvement of the CCR4-Not complex in transcription (Kruk et al., 2011). Human CNot2 regulates the deadenylase activity and structural integrity of the CCR4–Not complex (Russell et al., 2002), whereas yeast Not2p is thought to mediate intra-nuclear interactions between chromatin components and the transcriptional complex (Collart, 2003). In higher plants, the *Arabidopsis thaliana* genome has two highly similar Not2 homologs, AtNot2a and AtNot2b, which are required for *Agrobacterium tumefaciens*–mediated stable transformation and act as general factors to promote the transcription of protein coding and miRNA genes (Anand et al., 2007; Wang et al., 2013).

The CCR4-Not complex has been implicated in orchestrating gene expression networks that impact on virulence in human fungal pathogens such as *Candida albicans* and *Cryptococcus neoformans* (Panepinto et al., 2013). More recently, it was found that deletion of *FgNot2, FgNot3*, and *FgNot5* in *F. graminearum* resulted in reduced virulence toward wheat heads (Bui et al., 2016). The present study was initially to examine the biological function of *FonNot2* in *F. oxypsorum* f. sp. *niveum* (*Fon*), the causal agent of watermelon wilt disease. Our results showed that targeted disruption of *FonNot2* led to defects in mycelial growth, conidia production and morphology, virulence, cell wall integrity, fusaric acid (FA) production, oxidative stress tolerance and reactive oxygen species (ROS) production, demonstrating the importance of *FonNot2* in vegetative growth, conidiogenesis and virulence in *Fon*.

## Materials and Methods

### Strains and Growth Conditions

The *Fon* race 1 strain ZJ1, isolated from diseased watermelon from Zhejiang province of China, was used as wild type (WT) strain for fungal transformation, gene targeted disruption and complementation experiments in this study. For growth and stress tolerance assays, the tested *Fon* strains were cultivated on potato dextrose agar medium (PDA) (200 g/l potato and 20 g/l dextrose, pH7.0) at 26°C under constant fluorescent light. For conidiation assays, the tested *Fon* strains were cultivated in mung bean liquid (MBL) broth (30 g mung beans boiled in water for 20 min, filtered through cheesecloth and brought to 1 l).

### Bioinformatics Analysis

BLASTp was performed using human Not2 protein sequence (GenBank accession no. XP_011536702) as a query to search against downloaded local *F. oxysporum* genome database. Sequence alignment was carried out using ClustalW program in LaserGene software and phylogenetic tree was constructed using full-length Not2 proteins by neighbor-joining method in MEGA5 software with 1000 replications.

### Generation and Characterization of *FonNot2*-Targeted Disruption and Complementation Strains

Construction of *FonNot2*-targeted disruption vector was performed using double-joint PCR method (Yu et al., 2004). A 4.3 kb fragment including *FonNot2* coding sequence and putative 5′ and 3′ UTR sequences was used as target region for disruption. A 699 bp 5′ UTR fragment was amplified with primers FonNot2-5UTR-F and FonNot2-5UTR-R (containing a 28 bp overlapping HPH cassette sequence at 5′ end) (Supplementary Table S1); while a 646 bp 3′-UTR fragment was amplified with primers FonNot2-3UTR-F (containing a 28 bp overlapping HPH cassette sequence at 3′ end) and FonNot2-3UTR-R (Supplementary Table S1). A 1349 bp fragment containing the HPH cassette, which encodes hygromycin phosphotransferase under control of the *Aspergillus nidulans* TrpC promoter, was amplified from plasmid pBS-HPH with primers HPH-F and HPH-R (Supplementary Table S1). The amplified 5′ and 3′ UTR flanking sequences of the *FonNot2* gene were fused to the HPH cassette via double-joint PCR using primers FonNot2-5UTR-F and FonNot2-3UTR-R (Supplementary Table S1), yielding a 2694 bp fragment of the targeted disruption vector FonNot2-TD. Fungal protoplasts were generated from WT strain and were directly transformed with the targeted disruption vector FonNot2-TD after purification according to previously described method (Di Pietro and Roncero, 1998). Transformants were selected on PDA supplemented with 100 mg/l hygromycin (Hyg) and the resulting Hyg^R^ transformants were initially identified by PCR using primers FonNot2-JD-F and FonNot2-JD-R with genomic DNA prepared by CATB method (Luo et al., 2005). To construct the complementation vector, a 3515 bp fragment containing the native promoter and coding sequence of *FonNot2* was amplified from genomic DNA with primers FonNot2c-F and FonNot2c-R (Supplementary Table S1) and inserted into plasmid YF11-neo, yielding complementation vector pYF11-neo-FonNot2. Protoplasts were prepared from the ΔFonNot2 strain and were transformed with the complementation vector pYF11-neo-FonNot2. Transformants were selected on PDA with 50 mg/l neomycin and were confirmed by PCR using primers FonNot2C-JD-F and FonNot2C-JD-R (Supplementary Table S1) with genomic DNA prepared by CATB method (Luo et al., 2005). The obtained FonNot2-targeted disruption mutant ΔFonNot2 and complementation strain ΔFonNot2-C were purified via single spore isolation. For Southern blotting, genomic DNA was extracted from individual strain, digested completely with *Kpn*I, separated by electrophoresis on a 0.8% agarose gel, and transferred by capillary action overnight onto a Hybond-N^+^ membrane (Amersham Biosciences, Little Chalfont, UK) using 20 × SSC solution. A 805 bp 5′ UTR flanking fragment of *FonNot2* was amplified with primers FonNot2-TZ-F and FonNot2-TZ-R (Supplementary Table S1) and labeled with DIG by random priming method using a DIG High Prime DNA Labeling and Detection kit (Roche Diagnostics, Shanghai, China). Prehybridization, hybridization and detection were performed following the manufacturer’s recommendations.

### Growth and Developmental Phenotype, Cell Wall Sensitivity and Oxidative Stress Response Assays

Radial growth was measured on PDA and colony morphology was photographed after incubation at 26°C for 7 days. Conidia were harvested from 7-day-old cultures and counted with a haemacytometer. Microscopic observation of conidiophores was performed by placing hyphal blocks from 7-day-old cultures on glass cover slides for 24 h and then examined. Conidia harvested from 7-day-old cultures were added into YEPD (3 g yeast extract, 10 g peptone and 20 g dextrose in 1 l, pH 7.0) broth and incubated at 26°C on a rotary shaker at 180 rpm for 12 h. Percentage of conidial germination was determined by microscopic examination of at least 100 randomly selected conidia per field. For cellophane penetration assays, fungal colonies were grown for 3 days at 26°C on cellophane membranes placed on PDA and the cellophane membranes along with the fungal colonies were removed. The PDA plates were incubated for another 1 day to examine the presence of mycelial growth. Stress response assays were performed by inoculating 3-day-old mycelial plugs on PDA plates supplemented with 5 mM H_2_O_2_ or 3 mM paraquat for oxidative stress assays, or with 0.2% Calcofluor White (CFW) (Sigma–Aldrich, St. Louis, MO, USA) or 0.2% Congo Red (CR) (Sigma–Aldrich, St. Louis, MO, USA) for cell wall sensitivity assays. After incubation at 26°C for 7 days, radial colony growth was measured and percentage of inhibition was calculated by comparing the colony diameter under stressed condition with the diameter under normal condition.

### Inoculum Preparation, Pathogenicity Tests and Fungal Biomass Estimation

To prepare inoculum, colonized agar plugs from a 7-day-old PDA culture were transferred into a liquid MBL and grown for 4 days on a rotary shaker at 170 rpm at room temperature. Spores were collected by filtering through four layers of sterile cheesecloth and the spore suspension was adjusted to approximately 1 × 10^7^ spores/ml. Pathogenicity was tested by root-dip method using watermelon (*Citrullus lanatus* L.) cultivar Zaojia (a susceptible cultivar to race 1). Seedlings were grown in a vermiculite: plant ash: perlite (6:2:1) mixture in a growth room at 24°C with a cycle of 16 h light/8 h dark. Two-week-old seedlings were carefully uprooted and washed in tap water to remove soil particles. Roots of the seedlings were dipped for 30 s in spore suspensions prepared from WT, targeted disruption ΔFonNot2 and complementation ΔFonNot2-C strains. The inoculated seedlings were carefully replanted in the same growth medium and allowed for disease development. Disease scores were assessed 3 weeks after inoculation using the following rating scales: 0 = no symptom, 1 = yellowing, 2 = wilting and 3 = death. Fungal biomass measurement was performed as described previously (Thatcher et al., 2009). Root and stem samples were collected at 3, 6, and 9 days post-inoculation and the transcript levels of *Fon FonOpm12* and watermelon *ClRps10* genes were determined by qRT-PCR using a pair of *FonOpm12*-specific primers FonOpm12-F and FonOpm12-R and a pair of watermelon *ClRps10*-specific primers ClRps10-F and ClRps10-R, respectively (Supplementary Table S1). Relative fungal biomass was calculated by normalizing *FonOpm12* to watermelon *ClRps10* (Lin et al., 2010).

### Biochemical Measurements

Chitin content was measured according to a previously described method (Bulik et al., 2003). Briefly, ten *Fon* colonized agar plugs were inoculated into 200 ml YEPD liquid medium and incubated at 28°C on a rotary shaker at 180 rpm for 2 days. Mycelium was collected by filtering through three layers of Waterman filters and ground in liquid nitrogen. Five milligrams of the ground powder was resuspended in 1 ml 6% KOH and heated at 80°C for 90 min. After centrifugation at 12000 *g* for 10 min, the pellets were re-suspended in 1 ml 10 mM PBS (pH7.4) and the suspensions were re-centrifuged at 12000 *g* for 10 min. The resultant pellets were re-suspended in 100 μl Mcllvaine buffer (pH6.0) containing 5 μl chitinase from *Streptomyces plicatus* (Sigma–Aldrich, St. Louis, MO, USA) and incubated at 37°C for 16 h. One hundred microliters of the samples were combined with 100 μl 0.27 M sodium borate and heated for 10 min at 100°C. After cooling, 1 ml of freshly diluted (1:10) of DMAB reagent (10 g *p*-dimethylaminobenzaldehyde in 12.5 ml concentrated HCl and 87.5 ml glacial acetic acid, diluted 1:10 with glacial acetic acid) was added and incubated at 37°C for 20 min. The absorbance at 585 nm was recorded and the quantity of glucosamine in samples was calculated by referencing to a standard curve prepared with *N*-acetylglucosamine (Sigma–Aldrich, St. Louis, MO, USA). For quantification of FA content, fifteen *Fon* colonized agar plugs were inoculated into 200 ml Czapek Dox liquid medium and incubated at 28°C on a rotary shaker at 180 rpm for 15 days and the cultures were filtrated with Waterman filters to remove mycelium and conidia. After centrifugation at 4000 rpm for 10 min, the supernatant was adjusted to pH2.5 with 2 M HCl and extracted with ethyl acetate for three times. The combined ethyl acetate extractions were dried on a rotary evaporator and the resultant residues were dissolved in 5 ml methanol. A high performance liquid chromatography (HPLC) system (Model LC-20AD, Shimadzu Corporation, Kyoto, Japan) with an Agilent EclipseF XDB-C18 (4.6 × 250 mm, 5 μm) was employed to analyze FA content. Elution was carried out using a mobile phase comprising 30% acetonitrile and 70% H_3_PO_4_ (0.4%) for 20 min at flow rate of 1 ml/min with a UV detector at 280 nm. Before injection, the samples were filtrated through 0.45 μm filters. Quantification of FA in samples was done on the basis of the peak area compared to a standard curve prepared using the same procedure. For measurement of peroxidase (POD) activity, ten *Fon* colonized agar plugs were inoculated into 200 ml PDA liquid medium and incubated at 28°C on a rotary shaker at 180 rpm for 2 days. Culture filtrates were collected by passing through three layers of Waterman filters and 1 ml of the filtrates were centrifuged at 5000 *g* for 5 min. Five hundred microliters of the supernatants were added to 1.5 ml reaction mixture containing 50 mM acetate buffer (pH 5.0) and 10 mM ABTS and incubated at 25°C for 5 min with 3 mM H_2_O_2_. Absorbance at 420 nm was measured spectrophotometrically.

### Detection of ROS

For detection of ROS in vegetative mycelia, hyphae were collected from 2-day-old culture and stained with 1.5 mM 2′, 7′-dichlorodihydrofluorescein diacetate (DCFH-DA) (Beyotime Biotechnology, Nantong, China) in 50 mM phosphate buffer (pH8.5) for 20 min under dark conditions. After washing twice with the same buffer, the samples were observed for fluorescence using a Nikon eclipse Ni-U microscope with a selective GFP filter and digital images were captured with a Nikon DS-Fi1c CCD camera using NIS-Elements D45000 software (Nikon, Tokyo, Japan). For detection of superoxide anion, spores were harvested from 7-day-old cultures and allowed to germinate in YEPD broth at 26°C on a rotary shaker at 180 rpm for 12 h. Hyphae from 2-day-old culture or spores germinated for 12 h were stained with 0.05% (wt/vol) nitroblue tetrazolium (NBT) solution for 20 min. After stopping by the addition of ethanol, the formazan staining was observed under a Nikon eclipse Ni-U microscope using NIS-Elements D44000 software and digital images were captured by a Nikon DS-Fi2 camera (Nikon, Tokyo, Japan). Mean pixel intensity within regions of interest in the digital images of DCFH-DA-stained mycelia or NBT-stained mycelia and spores was calculated using Image Pro Plus 6.0 software. At least 20 representative mycelia or germinating spores were examined in an independent experiment.

### Scanning Electron Microscopy Observation

For SEM observation, 2-week-old seedlings were inoculated by dipping the roots into *Fon* spore suspension (1 × 10^7^ spores/ml) on a rotary shaker at 80 rpm and root samples were collected 24 h later. The samples were fixed overnight in phosphate buffer (pH7.0) with 2.5% glutaraldehyde, and then post-fixed with 1% osmic acid in phosphate buffer (pH7.0) for 1 h. After dehydration in an ethanol series, the samples were transferred to a mixture of alcohol and isoamyl acetate (v:v = 1:1) for 30 min and then transferred to pure isoamyl acetate for 1 h. The samples were dehydrated in Hitachi Model HCP-2 critical point dryer (Hitachi, Tokyo, Japan) with liquid CO_2_. Finally, the dehydrated samples were coated with gold-palladium and observed in Hitachi Model TM-1000 scanning electron microscope (Hitachi, Tokyo, Japan) at 15 kV.

### RNA Manipulation and Quantitative RT-PCR (qRT-PCR) Analysis

Fungal mycelial, conidial and germinating conidial samples or *Fon*- and mock-inoculated watermelon root samples were used for analysis of the *FonNot2* expression, whereas mycelial samples collected from 7-day-old cultures were used for analysis of the expression of genes associated with pathogenicity, toxin and chitin biosynthesis and oxidative stress. Total RNA was extracted using Trizol reagent (Invitrogen, Shanghai, China) and treated with RNase-free DNase (TaKaRa, Dalian, China). First-strand cDNA was synthesized from 1 μg of total RNA using AMV reverse transcriptase (TaKaRa, Dalian, China) according to the manufacturer’s recommendation. qRT-PCR was performed with three technical replicates and run on a CFX96 real-time PCR system (BioRad, Hercules, CA, USA). SYBR Premix Ex Taq kit (TaKaRa, Dalian, China) was used to prepare qRT-PCR reaction, which contained 10 μl 2 × SYBR Premix Ex Taq buffer, 1 μg synthesized cDNA and 10 μmol of each of gene-specific primers in a final volume of 20 μl. *FonActin* was used as an internal control to normalize the data for comparing the relative transcript abundance of the target genes. Relative expression of genes of interest was calculated using the 2^-ΔΔCT^ method. Gene-specific primers used in qRT-PCR are listed in Supplementary Table S1.

### Statistical Analysis

All experiments were repeated independently for three times and each experiment was set with three replicates. Data were statistically analyzed using Student’s *t*-test at *p* = 0.05 level.

## Results

### Characterization of *FonNot2*

**FIGURE 1:**
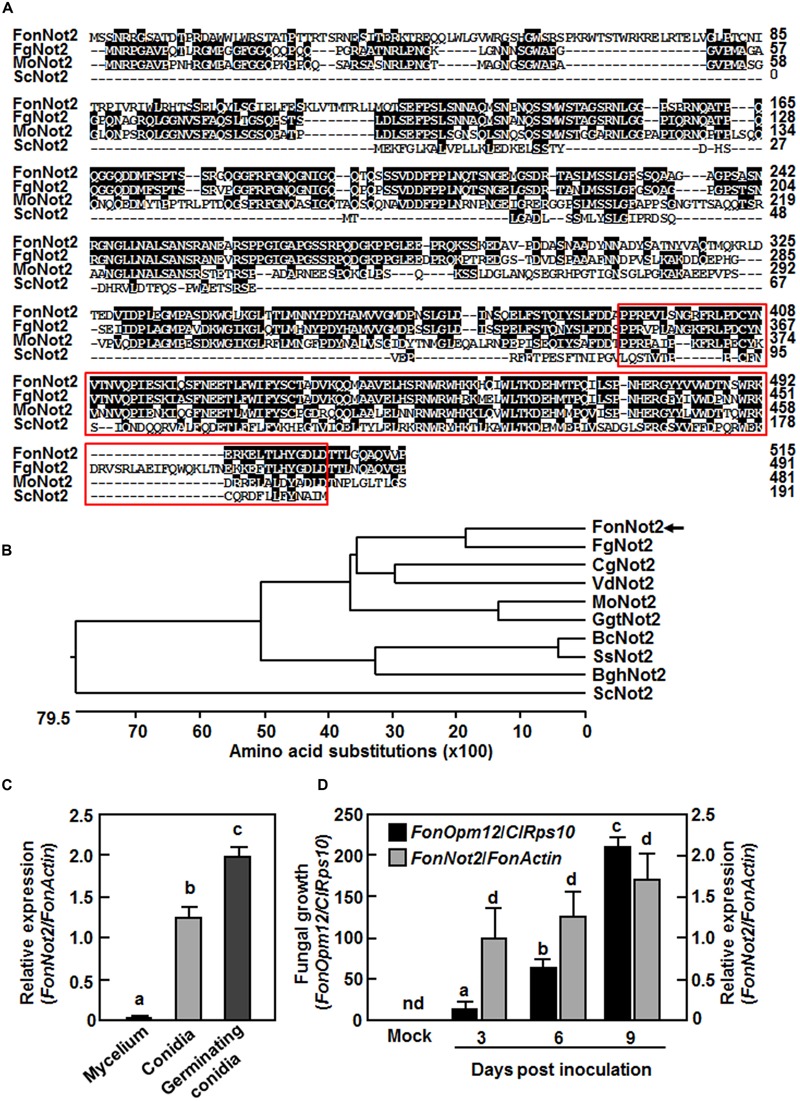
**Sequence characteristics of FonNot2 and expression profiles of *FonNot2*. (A,B)** Sequence alignment and phylogenetic tree of FonNot2 with homologs from other fungi. Putative Not2/3/5 domain is boxed with red line. FonNot2 is indicated by an arrow. Sequences used for alignment and tree construction are as follows: BghNot2, *Blumeria graminis* f. sp. *hordei* (CCU74264.1); BcNOT2, *Botrytis cinerea* (EMR84714.1); CgNot2, *Colletotrichum graminicola* (XP_008091790.1); FgNot2, *Fusarium graminearum* (ESU11019.1); GgtNot2, *Gaeumannomyces graminis* var. *tritici* (XP_009218872.1); MoNot2, *Magnaporthe oryzae* (XP_003719658.1); ScNot2, *Saccharomyces cerevisiae* (CAA48160.1); VdNot2, *Verticillium dahliae* (XP_009650328.1). **(C,D)** Expression profiles of *FonNot2* at different fungal developmental stages **(C)** and in infected watermelon roots **(D)**. Mycelium and conidia were collected from 10-day-old *Fon* cultures and germinating conidia were harvested at 6 h after incubation in YEPD. Two-week-old seedlings were inoculated with *Fon* spore suspension and root samples were collected at indicated time points. qRT-RCR data were normalized with the transcript level of *FonActin* and relative expression of *FonNot2* was shown as folds of the transcript level of *FonActin*. Fungal growth was shown as ratios of the transcript levels of *FonOpm12* and *ClRps10* genes*0*. Data presented in **(C,D)** are the means ± SD from three independent experiments and different letters above the columns indicate significant difference at *p* < 0.05 level. nd, not detectable.

To identify *FonNot2*, putative *F. oxysporum Not2* gene (FOMG_12536) was identified via BLASTp searching in the *F. oxysporum* genome database. A 2760 bp genomic fragment containing the *FonNot2* gene and a 1545 bp cDNA fragment was amplified and sequenced. Alignment of the genomic and cDNA sequences revealed that *FonNot2* contains one intron of 819 bp and that the entire coding sequence of *FonNot2* is 1548 bp, encoding a 515 amino acid protein. The FonNot2 protein contains a conserved NOT2/NOT3/NOT5 domain at its C-terminal (Collart and Struhl, 1994) (**Figure 1A**). Phylogenetic tree analysis indicated that FonNot2 is closely related to *F. graminearum* FgNOT2, *Colletotrichum graminicola* CgNOT2, *Verticillium dahliae* VdNOT2, *Magnaporthe oryzae* MoNOT2 and *Gaeumannomyces graminis* var. *tritici* GgtNOT2 (**Figure 1B**) and shows 71, 52, 48, 49, and 52% of amino acid sequence identity to the above-mentioned Not2 proteins from different pathogenic fungal species, respectively.

### *FonNot2* Is Highly Expressed in Conidia and Germinating Conidia and during Plant Infection Process

We first analyzed the expression profile of *FonNot2* in mycelia, conidia and germinating conidia and during root infection by qRT-PCR. In comparison to the expression in mycelia, the expression of *FonNot2* was significantly increased in conidia and showed a further increase in germinating conidia, resulting in >200-fold of increases over that in mycelia (**Figure 1C**). In mock-inoculated watermelon roots, no transcript of *FonNot2* was detected (**Figure 1D**). Over a 9-day period after *Fon* inoculation, *in planta* growth of *Fon* in roots of the inoculated plants increased gradually, as shown by the ratios of the transcript levels of *FonOpm12*/*ClRps10* (**Figure 1D**). Similarly, the expression level of *FonNot2* increased gradually with the progress of disease development in *Fon*-inoculated watermelon roots, as shown by the ratios of the transcript levels of *FonNot2*/*FonActin* (**Figure 1D**). The expression level of *FonNot2* in inoculated watermelon roots was significantly higher than that in mycelia of axenic cultures (**Figures 1C,D**), indicating that *FonNot2* could be induced during infection process. These data indicate that *FonNot2* is highly expressed in conidia and germinating conidia and during plant infection process.

### Generation of *FonNot2*-Targeted Disruption Mutant ΔFonNot2 and ΔFonNot2 Complementation Strain ΔFonNot2-C

**FIGURE 2:**
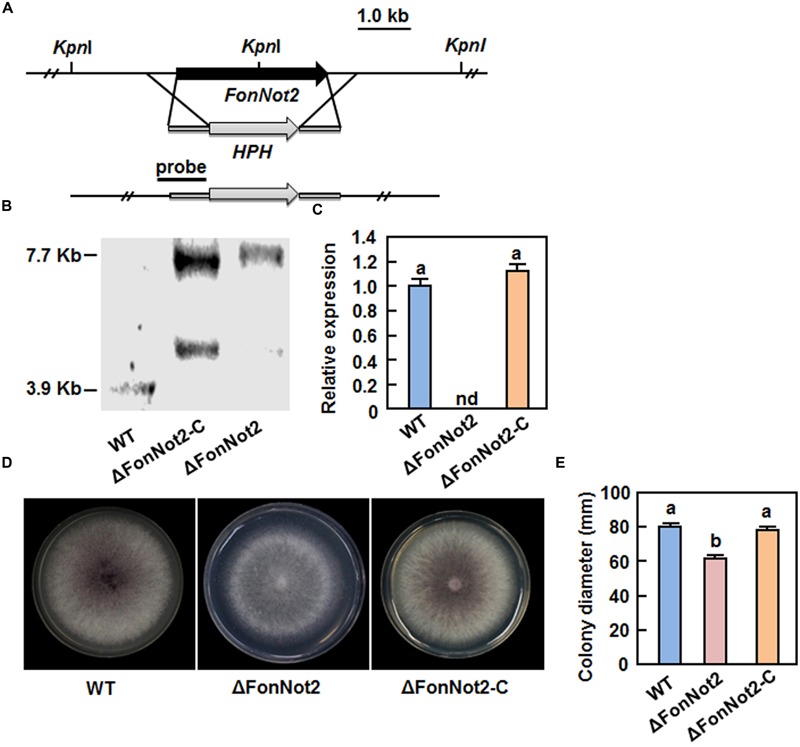
**Characterization and vegetable growth of *FonNot2*-targeted disruption mutant strain ΔFonNot2 and complementation strain ΔFonNot2-C. (A)** Schematic strategy used for generation of the targeted disruption strain ΔFonNot2. HPH, hygromycin B resistance gene cassette. The 5′- and 3′-flanking region (black bars) of the *FonNot2* ORF used as a probe for hybridization is indicated. **(B)** Southern blotting of the targeted disruption strain ΔFonNot2 and complementation strain ΔFonNot2-C. **(C)** Expression of *FonNot2* in axenic cultures of WT,ΔFonNot2 and ΔFonNot2-C strains. qRT-PCR data were normalized with the transcript level of *FonActin*. Relative expression of *FonNot2* in the WT strain was set to 1. nd, not detectable. **(D)** Mycelial growth and colony morphology of the WT,ΔFonNot2 andΔFonNot2-C strains on PDA plates. Photos were taken 7 days after incubation. **(E)** Inhibition of the radial growth of the WT,ΔFonNot2 and ΔFonNot2-C strains grown on PDA plates. Data presented in **(C,E)** are the means ± SD from three independent experiments and different letters above the columns indicate the significant difference at *p* < 0.05 level between WT, ΔFonNot2 and/or ΔFonNot2-C strains.

To explore the function of *FonNot2*, we generated a *FonNot2*-targeted disruption strain ΔFonNot2 by replacing a 4399 bp fragment containing the entire *FonNot2* ORF and partial 5′ and 3′ UTR sequences with the 2694 bp targeted disruption vector containing HPH cassette (**Figure 2A**). After protoplast transformation with the targeted disruption vector, 21 Hyg-resistant (Hyg^R^) transformants were obtained. Seven candidates of the targeted disruption mutants were initially screened from these Hyg^R^ transformants by PCR amplification of an expected 2015 bp fragment in disruption mutants instead of a 3720 bp fragment in WT strain. Southern blotting of genomic DNA probed with an 805 bp fragment in the 5′ UTR flanking region revealed that the 3.9 Kb *Kpn*I fragment in WT strain was replaced by a 7.7 Kb *Kpn*I fragment in the targeted disruption mutants (**Figure 2B**), demonstrating the targeted disruption of the *FonNot2* gene in the mutants, which was named ΔFonNot2. Because the successful *FonNot2*-targeted disruption strains had the same phenotype, only one ΔFonNot2 strain was chosen for further study. Complementation of the ΔFonNot2 strain was performed by introducing a 3515 bp DNA fragment encompassing the promoter and entire coding sequence of the *FonNot2* gene into the ΔFonNot2 strain. Several neomycin-resistant transformants were obtained and verified by PCR amplification of a fragment identical to that obtained from the WT strain but absent from the ΔFonNot2 strain. Southern blotting of genomic DNA probed with the 805 bp fragment in the 5′ UTR flanking region revealed that, in addition to a 7.7 Kb *Kpn*I fragment identical to that in the ΔFonNot2 strain, the obtained complementation transformant contained an additional *Kpn*I fragment, demonstrating that the complementation of the *FonNot2* disruption in the ΔFonNot2 strain, which were named ΔFonNot2-C. qRT-PCR analysis revealed that the expression of *FonNot2* in the ΔFonNot2 strain was undetectable while the expression level in the ΔFonNot2-C strain was similar to that in WT strain (**Figure 2C**), confirming that the obtained ΔFonNot2 strain is a null mutant of *FonNot2*.

### *FonNot2* Is Required for Vegetative Growth and Conidiogenesis But Is Not Essential for Conidial Germination

**FIGURE 3:**
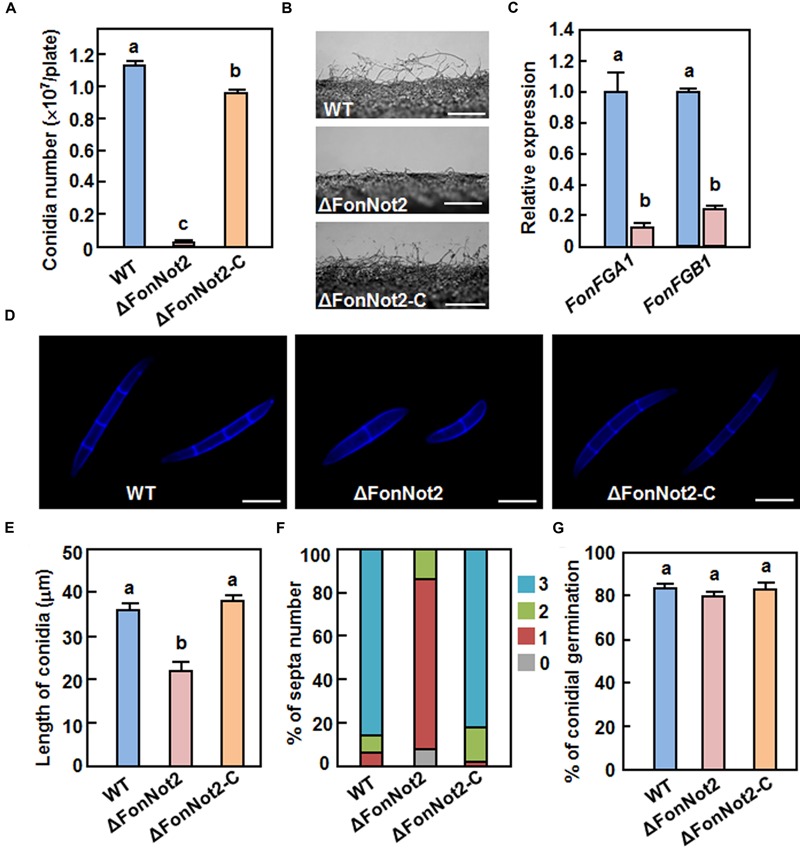
**Targeted disruption of *FonNot2* affects conidial morphology and conidia production but does not affect conidia germination in *F. oxysporum* f.sp.** *niveum*. **(A)** Conidia production. **(B)** Conidiophores. **(C)** Expression of conidiation-related genes. **(D)** Conidial morphology. **(E)** Conidial length. **(F)** Conidial septa number. **(G)** Conidial germination. Conidia were collected from 10-day-old cultures of the WT, ΔFonNot2 and ΔFonNot2-C strains grown in MBL. Conidial morphology, conidial length and septa number were examined by DIC. Conidia production was counted with a haematocytometer after incubation in MBL broth for 4 days in a shaker. Conidia were suspended in YEPD broth for 12 h and germination of 100 conidia was randomly examined. Total RNA was extracted 18 h after germination in PDA liquid medium and relative expression in the WT strain was set to 1. Data presented in **(A,C,E,G)** are the means ± SD from three independent experiments and different letters above the columns indicate the significant differences at *p* < 0.05 level between WT, ΔFonNot2 and/or ΔFonNot2-C strains.

To investigate the function of *FonNot2* in fungal growth and development, phenotype of the ΔFonNot2 strain was compared with the WT and ΔFonNot2-C strains. When grown on PDA, the ΔFonNot2 strain grew slowly, by a reduction of 23% in growth rate, and produced less pink pigment, as compared with WT strain (**Figures 2D,E**). The mycelial growth defect in the ΔFonNot2 strain was restored by complementation with *FonNot*2 (**Figures 2D,E**), which showed a growth rate and colony pigmentation similar to that of the WT strain. When grown in MBL, the ΔFonNot2 strain produced significantly less macroconidia, resulting in a reduction of 98% in comparison to that in WT strain (**Figure 3A**). Microscopic examination revealed that only a few conidiophores were observed in the ΔFonNot2 strain whereas typical conidiophores were normally developed in WT strain (**Figure 3B**). qRT-PCR analysis indicated that expression levels of *FonFGA1* and *FonFGB1*, encoding the heterotrimeric G protein subunits that are involved in conidiation in *F. oxysporum* (Jain et al., 2002, 2003), were significantly decreased in the ΔFonNot2 strain, as compared with those in WT strain (**Figure 3C**). Microscopic examination revealed that the macroconidia of the ΔFonNot2 strain lacked the typical morphology and were shorter, leading to a reduction of 39% in length, than those of the WT strain (**Figures 3D,E**). CFW staining assays showed that 80% of the macroconidia of the ΔFonNot2 strain had a single septum, and 8 and 12% of the macroconidia had 0 and 2 septa, respectively (**Figures 3D,F**). In contrast, 93% of the macroconidia from the WT strain had 3 septa (**Figures 3D,F**). The complementation strain ΔFonNot2-C showed indistinguishable changes in macroconidia morphology and conidiation ability as the WT strain (**Figures 3A,B,D–F**). The macroconidia produced by the ΔFonNot2 strain were able to germinate with similar germination rate to those of the WT and ΔFonNot2-C strains (**Figure 3G**). These results indicate that targeted disruption of *FonNot2* not only affects the vegetable growth but also influences conidiation and conidial morphology.

### *FonNot2* Is Required for Full Virulence on Watermelon and *In planta* Growth within Host Tissue

**FIGURE 4:**
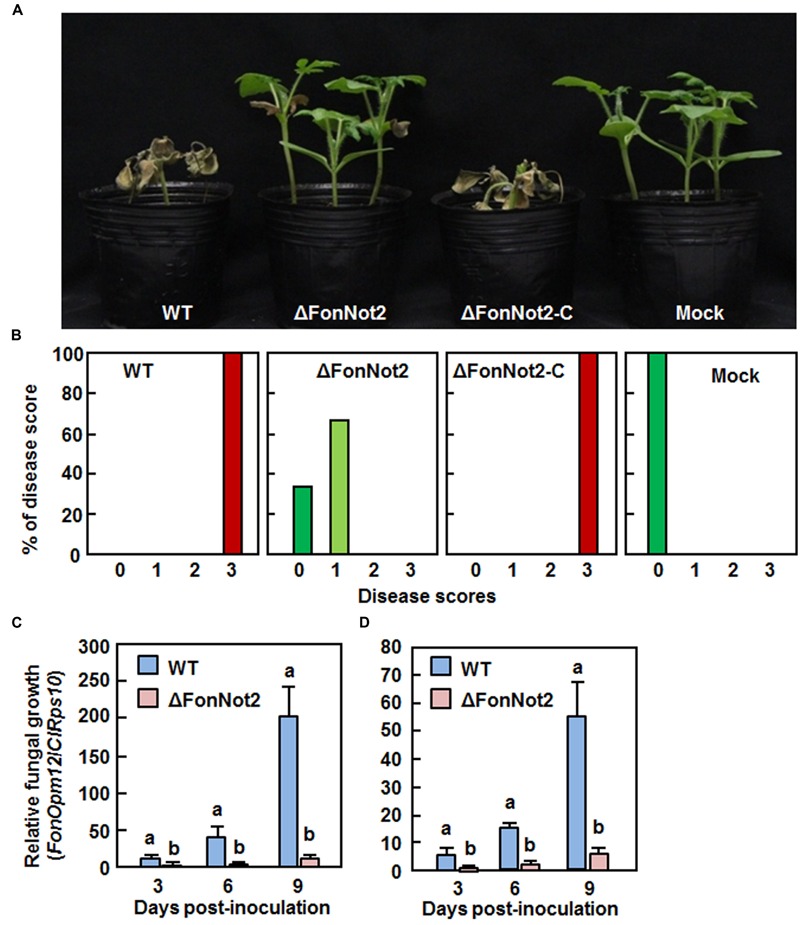
**Targeted disruption of *FonNot2* impairs virulence of *F. oxysporum* f.sp. *niveum* on watermelon.** Groups of 12 watermelon plants were inoculated by dipping the roots in spore suspension of the WT, ΔFonNot2 andΔFonNot2-C strains. **(A)** Disease phenotype and **(B)** disease severit in inoculated plants at 3 weeks after inoculation. Disease severity was assessed on a 4-scale rating standard. **(C,D)** *In planta* fungal growth in roots and stems of the inoculated plants. Relative *in planta* fungal growth was evaluated by qRT-RCR analysis of *FonOpm12* and watermelon *ClRps10* genes and shown as ratios of *FonOpm12*/*ClRps10*. Data presented in **(C,D)** are the means ± SD from three independent experiments and different letters above the columns indicate the significant difference at *p* < 0.05 level between WT and ΔFonNot2 strains at the same time point.

To examine the role of *FonNot2* in virulence, root infection assays were performed by dipping the roots of 2-week-old seedlings in conidial suspension of the WT, ΔFonNot2 and ΔFonNot2-C strains. In our repeated experiments, the ΔFonNot2-inoculated plants showed significantly reduced disease symptom development (**Figure 4A**), with 65 and 35% of the inoculated plants showing yellowing symptom on leaves or no obvious symptom at 3 weeks after inoculation (**Figure 4B**). By contrast, the WT- and ΔFonNot2-C-inoculated plants showed severe disease severity and all of the inoculated plants died at 3 weeks after inoculation (**Figures 4A,B**). To determine whether targeted disruption of *FonNot2* affected *in planta* fungal growth, we quantified fungal biomass in root and stem tissues by analyzing the transcript levels of *Fon FonOpm12* and watermelon *ClRps10* genes as an indicator and an internal reference, respectively. The fungal biomass in root and stem tissues of the WT strain-inoculated plants increased during 3–9 days post inoculation (**Figures 4C,D**); however, the fungal biomass in root and stem tissues of the ΔFonNot2-inoculated plants were significantly reduced at 3, 6, and 9 days post inoculation (**Figures 4C,D**), showing reduction of 20-fold in roots and 10-fold in stem at 9 days post inoculation. These data suggest that targeted disruption of *FonNot2* reduces the virulence of *Fon* on watermelon and weakens the ability of *Fon* to grow within watermelon tissues, demonstrating that *FonNot2* is required for full virulence toward watermelon.

### *FonNot2* Is Required for Successful Penetration on Watermelon Roots

**FIGURE 5:**
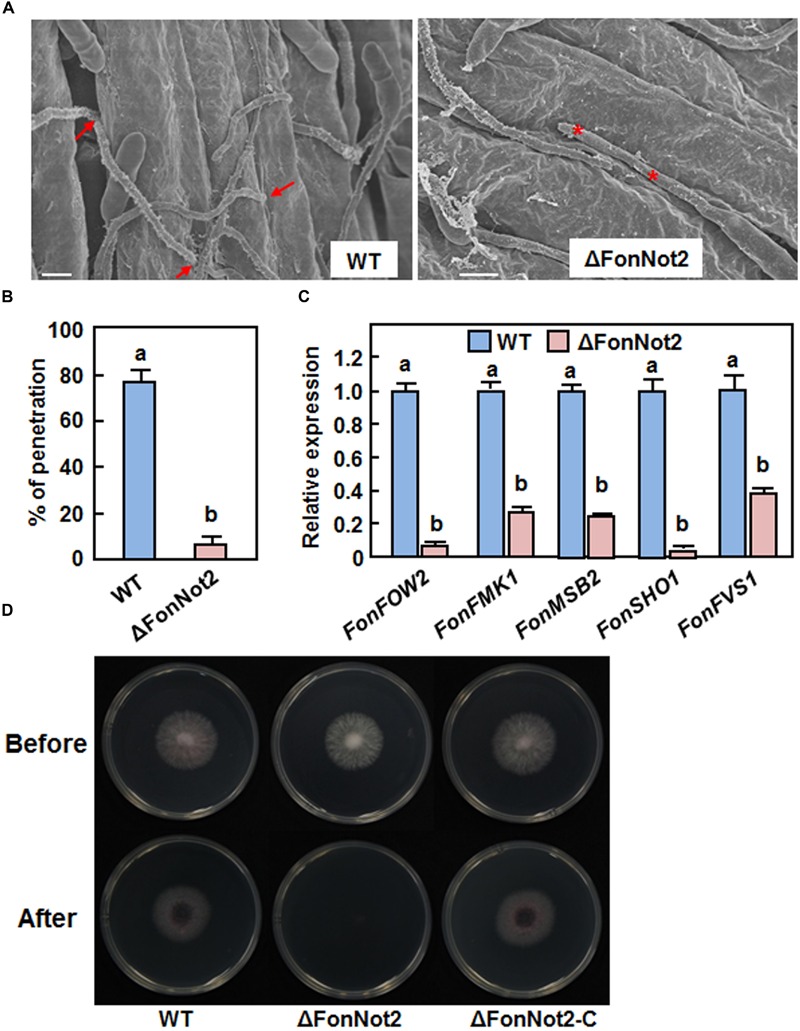
***FonNot2* is required for successful penetration of *Fon* to watermelon roots.** Watermelon plants were inoculated by dipping the roots into spore suspension of the WT and ΔFonNot2 strains and scanning electron microscopy analysis of watermelon roots was conducted at 24 h post inoculation. **(A)** Comparison of the infection behavior of the WT and ΔFonNot2 strains on watermelon roots. Arrows point to penetration events and asterisks indicate unsuccessful penetration events. **(B)** Penetration percentages of the WT and ΔFonNot2 strains on watermelon roots. At least 20 germinated conidia per strain were surveyed in each experiment. **(C)** Expression of infection-related genes in axenic cultures of the WT and Δ*FonNot2* strains. qRT-PCR data were normalized with the transcript level of *FonActin* and relative expression of the tested genes in WT strain was set to 1. **(D)** Comparison of the penetration ability against cellophane membranes. Fungal colonies were grown for 3 days on cellophane membranes placed on PDA (Before). The cellophane membranes with the fungal colonies were removed, and the plates were incubated for another 1 day to examine the presence of mycelial growth (After). Data presented in **(B,C)** are the means ± SD from three independent experiments and different letters above the columns indicate the significant difference at *p* < 0.05 level between WT and ΔFonNot2 strains.

We examined whether targeted disruption of *FonNot2* affected the penetration of *Fon* on watermelon roots. To test this hypothesis, watermelon roots inoculated with conidia of the WT and ΔFonNot2 strains were examined after 24 h by SEM. In the WT strain-inoculated roots, typical penetration events through openings at the junctions between epidermal cells were seen (**Figure 5A**, *left*) and 78% of the germinated conidia showed penetration event (**Figure 5B**). In the ΔFonNot2-inoculated roots, however, most of the germinated conidia grew on the root surface but failed to penetrate (**Figure 5A**, *right*) and only 8% of the germinated conidia showed penetration event (**Figure 5B**). We further compared the expression levels of some infection-related pathogenicity genes including *FonFOW2, FonFMK1, FonMSB2, FonSHO1* and *FonFVS1* (Di Pietro et al., 2001; Imazaki et al., 2007; Pérez-Nadales and Di Pietro, 2011, 2015; Iida et al., 2014) in axenic cultures of the WT and ΔFonNot2 strains. qRT-PCR analysis showed that the expression levels of these genes in the ΔFonNot2 strain were significantly reduced in comparison to the levels in the WT strain (**Figure 5C**). Furthermore, cellophane penetration assays were performed to confirm the defect in penetration ability of the ΔFonNot2 strain. As shown in **Figure 5D**, the ΔFonNot2 strain was unable to penetrate the cellophane membrane whereas the WT and complementation ΔFonNot2-C strains penetrated the cellophane membranes. These results indicate that targeted disruption of *FonNot2* weakens the ability of *Fon* to penetrate on watermelon roots, demonstrating that *FonNot2* contributes to penetration of *Fon* toward roots of watermelon plants.

### *FonNot2* Is Required for FA Biosynthesis

**FIGURE 6:**
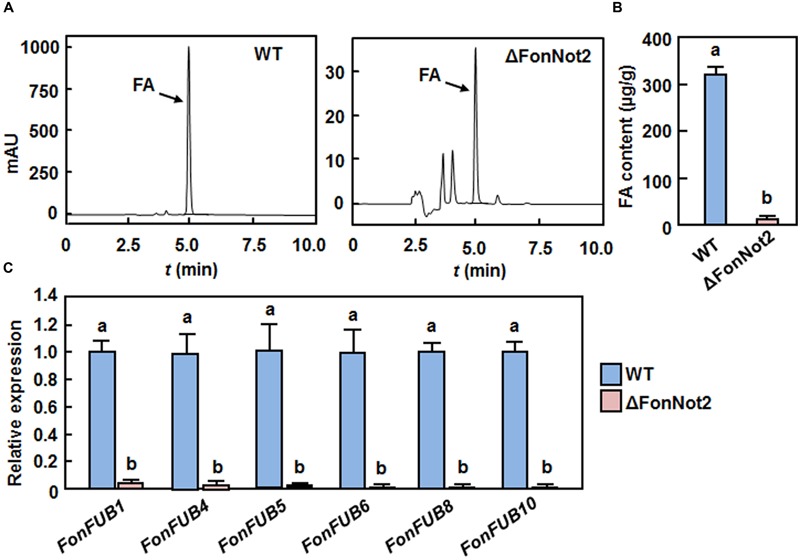
**Targeted disruption of *FonNot2* affects FA biosynthesis. (A)** HPLC chromatograms showing FA retention in cultures of the WT and Δ*FonNot2* strains. **(B)** FA levels in cultures of the WT and Δ*FonNot2* strains. **(C)** Expression levels of FA biosynthesis genes in axenic cultures of the WT and Δ*FonNot2* strains. qRT-PCR data were normalized with the transcript level of *FonActin* and relative expression of the tested genes in the WT strain was set to 1. Data presented in **(B,C)** are the means ± SD from three independent experiments and different letters above the columns indicate the significant difference at *p* < 0.05 level between WT and ΔFonNot2 strains.

FA, a polyketide-derived secondary metabolite, is thought to act as a mycotoxin contributing to severity of *F. oxysporum*-induced vascular wilt diseases (Brown et al., 2012, 2015). To examine whether targeted disruption of *FonNot2* affected the biosynthesis of FA, we compared the FA production in Czapek medium by the WT and ΔFonNot2 strains using HPLC method. Under our experimental condition, FA was detected in filtrates of the WT and ΔFonNot2 strains (**Figure 6A**). However, the amount of FA produced by the ΔFonNot2 strain was significantly decreased, resulting in a reduction of 94%, compared with that of the WT strain (**Figure 6B**). To confirm this, we further compared the expression levels of *FonFUB1, FonFUB4, FonFUB5, FonFUB6, FonFUB8* and *FonFUB10*, which are homologues of the FA-biosynthetic genes in *F. oxysporum* (Brown et al., 2015), in axenic cultures of the WT and ΔFonNot2 strains. As shown in **Figure 6C**, the expression level of all these FA biosynthetic genes in the ΔFonNot2 strain was markedly reduced, showing less than 10% of the levels in the WT strain. These results indicate that targeted disruption of *FonNot2* affects the FA biosynthetic pathway in *Fon*, suggesting that *FonNot2* plays a role in regulation of FA production.

### *FonNot2* Is Required for Maintenance of Cell Wall Integrity

**FIGURE 7:**
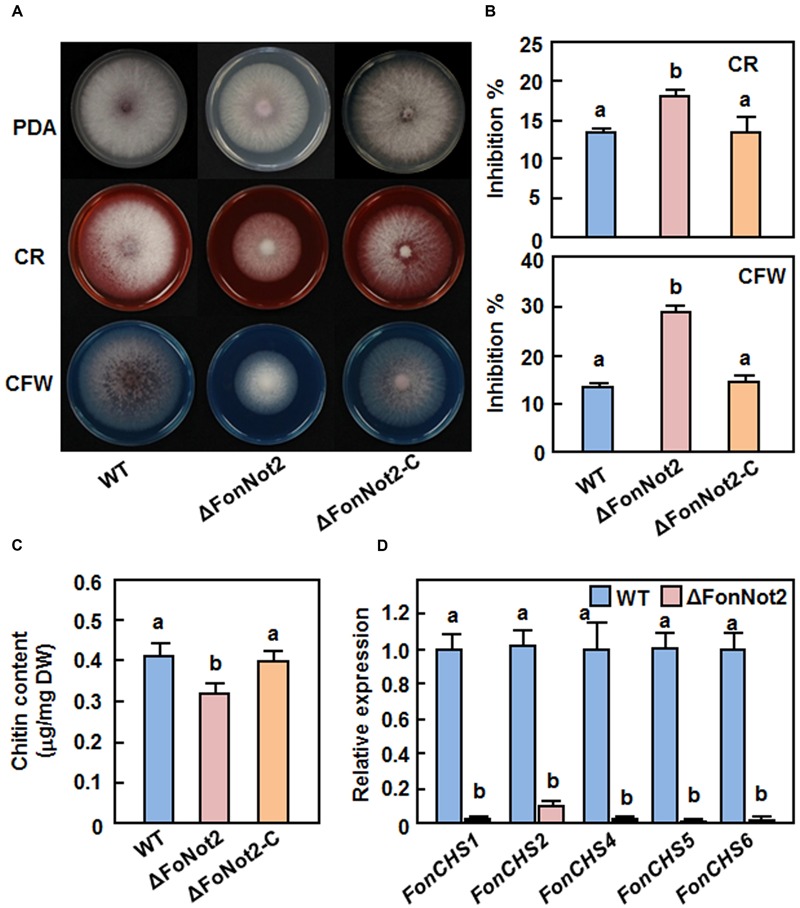
**Targeted disruption of *FonNot2* affects cell wall integrity of *F. oxysporum* f. sp. *niveum*. (A)** Mycelial growth and colony morphology. **(B)** Inhibition rates of the radial growth. The WT, ΔFonNot2 and ΔFonNot2-C strains were grown on PDA plates supplemented with or without CR (200 μg/ml) or CFW (200 μg/ml) and incubated at 26°C for 7 days. **(C)** Chitin content. **(D)** Expression levels of chitin synthase genes in axenic cultures of the WT and Δ*FonNot2* strains. Mycelial samples were collected from 7-day-old cultures for analysis of chitin content and gene expression. qRT-PCR data were normalized with the transcript level of *FonActin* and relative expression of the tested genes in the WT strain was set to 1. Data presented in **(B**,**C,D)** are the means ± SD from three independent experiments and different letters above the columns indicate the significant difference at *p* < 0.05 level between WT, ΔFonNot2 and/or ΔFonNot2-C strains.

To determine whether *FonNot2* has a function in maintaining cell wall integrity, we compared the mycelial growth of the WT, ΔFonNot2 and ΔFonNot2-C strains on PDA supplemented with or without 0.2% CR or 0.2% CFW, which are well-known fungal cell wall perturbing agents. After 7 days incubation, the ΔFonNot2 strain showed an extremely small colony in comparison to that of the WT strain on CR or CFW plates (**Figure 7A**). The mycelial growth inhibition rates of the ΔFonNot2 strain were increased by 35 and 116% on CR and CFW plates, respectively, as compared with those in the WT strain (**Figure 7B**), indicating the ΔFonNot2 strain was hypersensitive to cell wall perturbing agents. Considering that chitin is one of the major components in *F. oxysporum* cell wall (Schoffelmeer et al., 1999) and both of CR and CFW can inhibit fungal cell wall assembly by binding chitin, the chitin contents in the WT, ΔFonNot2 and ΔFonNot2-C strains were also compared. As shown in **Figure 7C**, the ΔFonNot2 strain showed a significantly reduced chitin content, leading to a reduction of 25%, as compared with those in the WT strains. The sensitivity to cell wall perturbing agents and the chitin content in the ΔFonNot2-C strain were comparable to those of the WT strain (**Figures 7A–C**). Because chitin synthases (CHS) are the enzymes responsible for chitin synthesis in *F. oxysporum* (Madrid et al., 2003; Martín-Udíroz et al., 2004; Martín-Urdíroz et al., 2008), we further compared the expression levels of *FonCHS1, FonCHS2, FonCHS4, FonCHS5* and *FonCHS6* in the WT and ΔFonNot2 strains. As expected, the expression levels of these tested *CHS* genes in the ΔFonNot2 strain were significantly downregulated, showing less than 10% of the levels in the WT strain (**Figure 7D**). These results indicate that targeted disruption of *FonNot2* affects chitin biosynthesis and that the reduced chitin content in the ΔFonNot2 strain is due to the downregulated expression of the *CHS* genes, suggesting that *FonNot2* plays a role in regulation of chitin biosynthesis in *Fon*.

### *FonNot2* Is Required for Oxidative Stress Tolerance and Affects ROS Production

**FIGURE 8:**
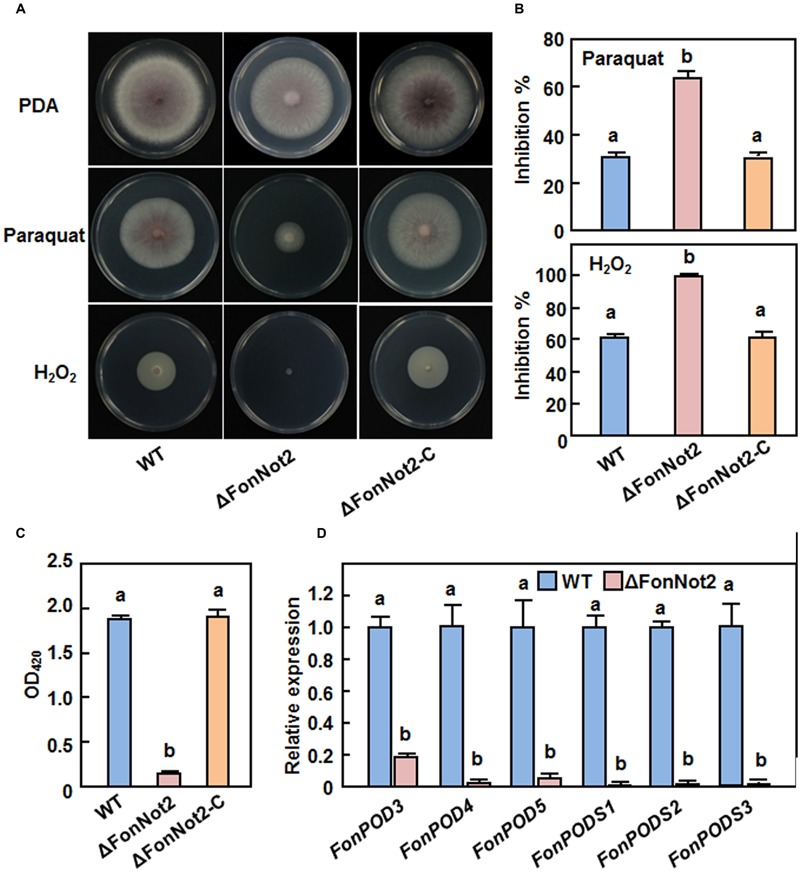
**Targeted disruption of *FonNot2* leads to increased oxidative sensitivity. (A)** Mycelial growth and colony morphology. **(B)** Inhibition rates of the radial growth. The WT, ΔFonNot2 andΔFonNot2-C strains were grown on PDA plates supplemented with or without 5 mM H_2_O_2_ and 3 mM paraquat and incubated at 26°C for 7 days. **(C)** Activity of extracellular peroxidase in culture filtrates. **(D)** Expression levels of *FonPOD* and *FonPODS* genes in axenic cultures of the WT and targeted disruption Δ*FonNot2* strains. Enzyme activity was measured by the ABTS oxidization test with H_2_O_2_. qRT-PCR data were normalized with the transcript level of *FonActin* and relative expression of the tested genes in the WT strain was set to 1. Data presented in **(B,C,D)** are the means ± SD from three independent experiments and different letters above the columns indicate the significant differences at *p* < 0.05 level between WT, ΔFonNot2 and/or ΔFonNot2-C strains.

It was recently shown that the production of ROS and/or detoxification of ROS are required for virulence in some phytopathogenic fungi including *F. oxysporum* (Molina and Kahmann, 2007; Chi et al., 2009; Qi et al., 2013). To examine whether *FonNot2* has a function in oxidative stress response, we compared the mycelial growth of the WT, ΔFonNot2 and ΔFonNot2-C strains on PDA supplemented with or without 5 mM H_2_O_2_ or 3 mM paraquat. As shown in **Figure 8A**, the ΔFonNot2 strain showed an extreme hypersensitivity to exogenous H_2_O_2_ and paraquat, as compared to the WT strain. The mycelial growth inhibition rates of the ΔFonNot2 strain were increased by 63 and 108% in the presence of H_2_O_2_ and paraquat, respectively, over those in the WT strain (**Figure 8B**). Accordingly, the extracellular POD activity in culture filtrates of the ΔFonNot2 strain was decreased, showing 7% of the activity in the WT strain (**Figure 8C**). The sensitivity to H_2_O_2_ and paraquat and the extracellular POD activity in the ΔFonNot2-C strain were comparable to those of the WT strain (**Figures 8A–C**). Considering that POD and peroxidase synthase (PODS) are effective enzymes in the ROS-detoxification systems, we thus compared the expression levels of three *FonPODs* (*FonPOD3, FonPOD4* and *FonPOD5*) and three *FonPODSs (FonPODS1, FonPODS2* and *FonPODS3*) in axenic cultures of the WT and ΔFonNot2 strains. As expected, the expression levels of the *FonPODs* and *FonPODSs* in the ΔFonNot2 strain were significantly downregulated, showing less than 20% of the levels in the WT strain (**Figure 8D**). These results indicate that targeted disruption of *FonNot2* weakens the oxidative stress tolerance and that the hypersensitivity of the ΔFonNot2 strain to oxidative stress is due to the downregulated expression of the genes involved in the ROS-detoxification systems, suggesting a role for *FonNot2* in regulation of the degradation of extracellular ROS.

**FIGURE 9:**
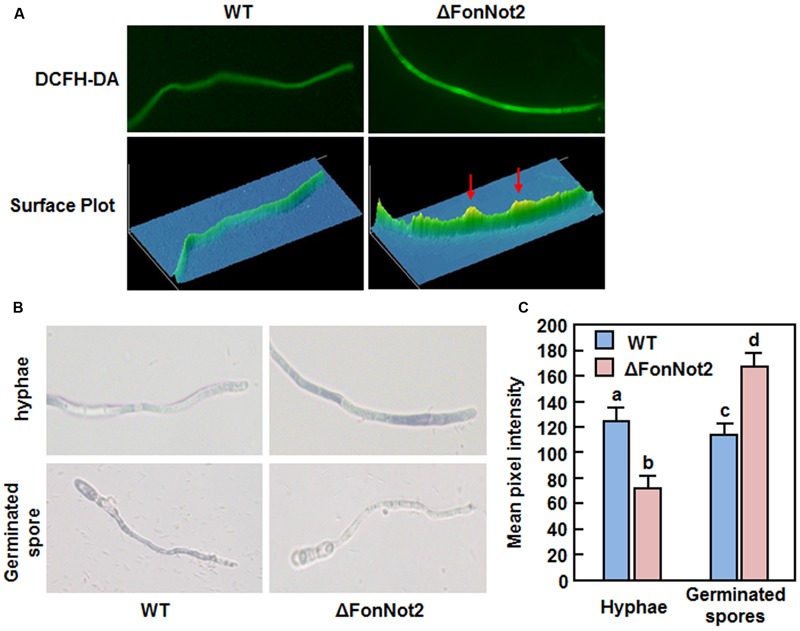
**Targeted disruption of *FonNot2* affects ROS production in vegetative myclia and germinating spores. (A)** ROS production in mycelia. Hyphae from 2-day-old culture were stained with DCFH-DA and images were obtained by examination under a fluorescent microscope. Surface plots were constructed using Image Pro Plus 6.0 software. **(B)** Production of superoxide anion in vegetative mycelia and germinating spores. Hyphae from 2-day-old culture and spores germinated for 12 h were stained with NBT and images showing formazan staining were captured by microscopic examination. **(C)** Mean pixel intensity in NBT-stained hyphae or spores. Data presented are the means ± SD from 20 measurements and columns with different letters indicate the significant difference at *p* < 0.05 level between WT and ΔFonNot2 strains.

We further investigated whether disruption of *FonNot2* affects the production of endogenous ROS by comparing the levels of ROS and superoxide anion in vegetative mycelia and germinating spores of the WT and ΔFonNot2 strains using DCFH-DA and NBT staining, respectively. Results from fluorescence detection and surface plot analysis indicate that the hyphae of the ΔFonNot2 strain produced more ROS than WT (**Figure 9A**). Similarly, examination of blue formazan precipitate produced from the reduction of NBT by superoxide anion, and quantitative analysis of mean pixel intensity revealed a significant increase in production of superoxide anion in hyphae of the ΔFonNot2 strain than that in WT (**Figures 9B,C**). Surprisingly, microscopic examination and analyses of mean pixel intensity revealed that the blue formazan precipitate accumulated significantly in germinating spores of WT strain but only less blue formazan precipitate in germinating spores of the ΔFonNot2 strain (**Figures 9B,C**). This result indicates that, during spore germination, germinating spores and the germ tubes of the ΔFonNot2 strain produced much less superoxide anion than WT spores. Taken together, these data suggest that disruption of *FonNot2* has differential effects on endogenous ROS production in vegetative mycelia and germinating spores in *Fon*.

## Discussion

Although the function of the CCR4-Not complex has been demonstrated in yeast and some human pathogenic fungi (Panepinto et al., 2013), less is known in phytopathogenic fungi. In this study, we characterized *FonNot2* in *Fon* and explored its functions in pathogenesis. Analysis of targeted disruption strain ΔFonNot2 revealed that loss of *FonNot2* function resulted in multiple defects in mycelial growth, conidia production and morphology, virulence, cell wall integrity, FA production, oxidative stress response and ROS production. Our data demonstrate the importance of FonNot2, a core subunit of the CCR4-Not complex, in regulation of vegetable growth, development and virulence in *F. oxysporum*.

The CCR4-Not complex has been implicated in fungal morphogenetic development (Panepinto et al., 2013). The reduced mycelial growth rate (**Figures 2C,D**) and decreased conidia production (**Figure 3A**) in the ΔFonNot2 strain suggest the involvement of *FonNot2* in vegetable growth and conidiation in *Fon*. The function of *FonNot2* in conidiogenesis was further supported by the significant down-regulation of *FonFGA1* and *FonFGB1*, two conidiation-related genes in *F. oxysporum* (Jain et al., 2002, 2003), in the ΔFonNot2 strain (**Figure 3C**). Similar phenotypes were also observed in *F. graminearum*, in which deletion of subunits in the CCR4-Not complex including *FgNot2* led to retarded vegetative growth and reduced conidia production (Bui et al., 2016). In addition, the macroconidia produced by the ΔFonNot2 strain showed abnormal morphology such as shorter in size and less septum number (**Figures 3D–F**), similar to the macroconidial morphology observed in *F. graminearum* strains with deletion of *FgNot2* (Bui et al., 2016). These data indicate that subunits of the CCR4-Not complex may have conserved functions in maintenance of normal macroconidial morphology in different *Fusarium* species. However, the macroconidia produced by the ΔFonNot2 strain germinated normally (**Figure 3G**), similar to the observation that conidia of the ΔFonNot2 strain germinated into germlings on watermelon roots as those of the WT (**Figure 5A**). By contrast, it was reported that disruption of *FgNot3* led to a significant reduction in conidia germination in *F. graminearum* (Bui et al., 2016). It is thus likely that functions of different subunits of the CCR4-Not complex may vary considerably in some growth and developmental stages of different fungal species.

We observed that the ΔFonNot2 strain showed a significantly decreased ability to cause wilt disease in watermelon (**Figure 4A**). Notably, ∼65% of the ΔFonNot2-inoculated watermelon plants displayed disease symptom with yellowing leaves (**Figure 4A**). These observations indicate that targeted disruption of *FonNot2* did not abolish the pathogenicity; instead, reduce the virulence, demonstrating a crucial function of *FonNot2* in virulence of *Fon*. Similar observation was also obtained in *F. graminearum*, in which deletion of *FgNot2, FgNot3* and *FgNot4* resulted in decreased virulence to wheat heads (Bui et al., 2016). During the *F. oxysporum*-plant interactions, germination of conidia in response to root exudate is the first and critical step toward infection of plant roots (Lagopodi et al., 2002). The facts that *FonNot2* was expressed at a higher level in germinating conidia than that in mycelia (**Figure 1C**) and its expression was upregulated during infection in watermelon roots (**Figure 1D**) suggest that *FonNot2* may specifically regulate some infection-related biological processes. However, conidia germination, hyphal elongation and attachment of germlings to watermelon roots in the ΔFonNot2 strain were comparable to that in the WT strain within 24 h after inoculation (**Figure 5A**), indicating that *FonNot2* may play a limited role in the early stages of infection. SEM observations of the inoculated roots showed that the ΔFonNot2 strain is defective in the ability to penetrate the root tissue (**Figures 5A,B**), which is associated with the downregulated expression of some infection-related genes including *FonFOW2, FonFMK1, FonMSB2, FonSHO1* and *FonFVS1* in the ΔFonNot2 strain (**Figure 5C**). These infection-related genes were previously shown to play key roles in penetration process of *F. oxysporum*, as deletion of each of them abolished the penetration ability to host roots (Di Pietro et al., 2001; Imazaki et al., 2007; Pérez-Nadales and Di Pietro, 2011, 2015; Iida et al., 2014). It is thus likely that *FonNot2* has an important function that is required for penetration of watermelon roots by *Fon*. On the other hand, a significant decrease in *in planta* fungal growth was observed in the ΔFonNot2 strain-inoculated plants (**Figures 4C,D**). Because of the reduced ability to penetrate the host roots in the ΔFonNot2 strain, a decreased amount of initial invading ΔFonNot2 strain within the inoculated roots should account for, at least partially, the reduced fungal biomass in the ΔFonNot2-inoculated plants. Collectively, we conclude that *FonNot2* is essential for full virulence of *Fon* on watermelon plants through affecting the penetration ability. However, it cannot be excluded that the reduced virulence of the ΔFonNot2 strain could be at least partially linked to defect in the cell wall integrity or to additional yet unknown pathways.

Phytopathogenic fungi in the genus *Fusarium* have been found to produce a wide variety of toxic secondary metabolites with different structure and biological activity, such as FA. Recently, a 12-gene FA biosynthetic gene cluster, coding for biosynthetic enzymes, two Zn(II)2Cys6 transcription factors and a transporter protein, was identified in *Fusarium* spp. including *F. oxysporum* (Brown et al., 2012, 2015; Niehaus et al., 2014; Crutcher et al., 2015). In the present study, we observed that the ΔFonNot2 strain produced significant less FA in the culture filtrates than the WT strain (**Figures 6A,B**) and this reduced FA production in the ΔFonNot2 strain was due to the downregulated expression of FA biosynthetic genes (**Figure 6C**). Similar observation was also obtained in *F. oxysporum* f.sp. *cubense*, in which deletion of each of three MPK genes *FoSlt2, FoMkk2* and *FoBck1* significantly affected the FA biosynthetic pathway, leading to reduced FA production (Ding et al., 2015). The role of *FonNot2* in FA biosynthesis differs from the observation that some of the subunits of the CCR4-Not complex including *FgNot2* are negative regulators of zearalenone and trichothecene production in *F. graminearum* (Bui et al., 2016). The function of FA produced by *F. oxypsorum* acts largely as a phytotoxin to disturb the physiological and metabolic processes in host plants (Dong et al., 2012), which accelerate the development of wilting symptom in infected plants. This is illustrated by the observations that lack of FA production did not affect virulence of *F. oxysporum* on its host plants (Brown et al., 2015). In this regard, it is possible that FA has a subsidiary role in *Fon* virulence to promote symptom development, as a coincidence between the reduced FA content (**Figure 6**) and decreased virulence on watermelon (**Figure 4**) in the ΔFonNot2 strain was observed. Furthermore, it was recently found that beauvericin, another mycotoxin produced by *F. oxysporum*, is a virulence factor on plant and mammalian hosts (López-Berges et al., 2013). Whether *FonNot2* is also involved in biosynthesis of beauvericin and whether this mycotoxin plays a role in *Fon* virulence on watermelon need to be further investigated.

Early studies in *Saccharomyces cerevisiae* and human fungal pathogens *C. albicans* and *C. neoformans* have shown that mutations in some subunits of the CCR4-Not complex relates to defect in the cell wall integrity (Kaeberlein and Guarente, 2002; Panepinto et al., 2007; Dagley et al., 2011). Similarly, we found that targeted disruption of *FonNot2* affects the cell wall integrity of *Fon*, as the ΔFonNot2 strain displayed an extreme hypersensitivity to cell wall perturbing agents such as CFW and CR (**Figures 7A,B**). However, this is contrast with the observation that deletion of *FgNot3* did not affect the cell wall integrity in *F. graminearum* (Bui et al., 2016). Chitin is a major cell wall component accounting for 10–20% of the total dry weight in filamentous fungi and is critical to maintain the cell wall integrity (Bartnicki-Garcia, 1968). An intact cell wall structure has been implicated in the complex infection process of *F. oxysporum* (Schoffelmeer et al., 1999) and mutations in some of the *CHS* genes led to defect in cell wall structure and loss of pathogenicity of *F. oxysporum* on their host plants (Madrid et al., 2003; Martín-Udíroz et al., 2004; Martín-Urdíroz et al., 2008). Defect in the cell wall integrity in the ΔFonNot2 strain is partially caused by the reduced chitin content, resulted from the downregulated expression of the *CHS* genes (**Figure 7**). It is likely that *FonNot2* functions in virulence to watermelon through affecting the cell wall integrity via a regulation on the expression of *CHS*s.

ROS play multiple roles in interactions between plants and microbes, either as host defense mechanisms (Lehmann et al., 2015) or as mediators of fungal pathogenesis (Heller and Tudzynski, 2011; Viefhues et al., 2014). *Arabidopsis* cell suspension cultures produced a significant ROS burst in response to an elicitor of *F. oxysporum* (Davies et al., 2006), implying the involvement of ROS burst during plant-*F. oxysporum* interaction. Due to the toxicity of ROS molecules, detoxification of the extracellular ROS is critical for *F. oxysporum* to achieve successful colonization within host plants (Heller and Tudzynski, 2011). We found that the ΔFonNot2 strain showed an extreme hypersensitivity to oxidative stress caused by exogenous H_2_O_2_ and paraquat (**Figure 8**), implying loss of the ability to degrade exogenous oxidative agents including H_2_O_2_. This differs from the observation that deletion of *FgNot3* did not affect the oxidative stress response in *F. graminearum* (Bui et al., 2016). H_2_O_2_ is generally scavenged by some antioxidant enzymes such as peroxidase, including ascorbate and glutathione peroxidases (Molina and Kahmann, 2007; Chi et al., 2009). Accordingly, we observed that targeted disruption of *FonNot2* led to downregulated expression of the *FonPOD* and *FonPODS* genes and thereby resulted in a dramatic decrease in POD activity in the ΔFonNot2 strain (**Figure 8**). These data imply that the decreased ability to degrade exogenous oxidative agents may be one of the mechanisms that are responsible for the reduced virulence of the ΔFonNot2 strain. This is in agreement with the observations that targeted disruption of each of three MPK genes or a bZIP transcription factor gene in *F. oxysporum* f. sp. *cubense* resulted in hypersensitivity to oxidative agents and reduced virulence to banana roots (Qi et al., 2013; Ding et al., 2015). It is thus likely that *F. oxysporum* possess similar mechanism to that of other phytopathogenic fungi such as *Maganporthe oryzae* and *Ustilago maydis* (Molina and Kahmann, 2007; Chi et al., 2009) to regulate oxidative stress response so that they counteract the extracellular ROS produced by host plants during pathogenesis. On the other hand, the decreased ROS-scavenging ability (**Figure 8**) may be one of the causes that lead to high levels of ROS including superoxide anion in vegetative mycelia of the ΔFonNot2 strain (**Figures 9A,B**). However, germinating spores of the ΔFonNot2 strain produced less ROS than WT (**Figures 9B,C**). This observations indicate that *FonNot2* may differentially affect the ROS production in vegetative mycelia and during spore germination process and that, in combination with the decreased penetration ability and reduced virulence in the ΔFonNot2 strain (**Figures 4** and **5**), ROS production during spore germination is essential for virulence in *Fon*. Similar observations were previously reported in *M. oryzae*, in which disruption of two superoxide-generating NADPH oxidase-encoding genes significantly increased ROS accumulation in mycelia but markedly reduced the ROS production during spore germination and appressorium development, leading to loss of pathogenicity on rice (Egan et al., 2007).

## Conclusion

We characterized *FonNot2*, encoding a core subunit of the CCR4-Not complex in *F. oxysporum*, and provided several lines of evidence supporting the important functions of *FonNot2* in regulating vegetable growth, conidiogenesis and conidia morphology, and virulence on watermelon. Further, the mechanism of *FonNot2* in virulence is related to its effects on cell wall integrity, oxidative stress response, ROS production and FA biosynthesis through the regulation of transcription of genes involved in multiple pathways. Recent studies have showed that the CCR4-Not complex acts in mRNA metabolism (Collart and Panasenko, 2012), in which Not2 serves as a general factor to promote transcription of protein-coding and non-coding genes in yeast and *Arabidopsis thaliana* (Kruk et al., 2011; Wang et al., 2013). Global effects of *FonNot2* on gene transcription and the involvement of other core subunits in the CCR4-Not complex in *F. oxysporum* virulence are open questions to be further investigated.

## Author Contributions

Conceived and designed the experiments: FS and YD. Performed the experiments: YD, ZC, LH, SL, ZS, YW, HW, HZ, and DL. Analyzed the data: FS and YD. Wrote the paper: FS.

## Conflict of Interest Statement

The authors declare that the research was conducted in the absence of any commercial or financial relationships that could be construed as a potential conflict of interest.
