# Supplementary material for: CCR4-Not Complex Subunit Not2 Plays Critical Roles in Vegetative Growth, Conidiation and Virulence in Watermelon Fusarium Wilt Pathogen Fusarium oxysporum f. sp. niveum
Source: Front Microbiol. 2016 Sep 16;7:1449. doi: 10.3389/fmicb.2016.01449 (PMC5025516; doi:10.3389/fmicb.2016.01449)
Supplement: Supplementary file 1 [file Table_1.DOC]

Supplementary Material

CCR4-Not Complex Subunit Not2 Plays Critical Roles in Vegetative Growth, Conidiation and Virulence in Watermelon Fusarium Wilt Pathogen *Fusarium oxysporum* f. sp. *niveum*

Yi Dai, Zhongye Cao, Lihong Huang, Shixia Liu, Zhihui Shen, Yuyan Wang, Hui Wang, Huijuan Zhang, Dayong Li and Fengming Song*

*** Correspondence:** Fengming Song, Email: fmsong@zju.edu.cn

# Supplementary Table

**Table S1: Primers used in this study with different purposes.**

| Genes | Accession No. or ID | Primer names | Sequence (5’-3’) |
| --- | --- | --- | --- |
| HPH |  | HPH-F | GGAGGTCAACACATCAATGCCTATT |
|  |  | HPH-R | CTACTCTATTCCTTTGCCCT |
| *Not2* | FOMG_12536 | FonNot2-5UTR-F | AACTCAACCAGGCCAAAGAG |
|  |  | FonNot2-5UTR-R | CAAAATAGGCATTGATGTGTTGACCTATAGGCAAGAAGGTAAGCA |
| *Not2* | FOMG_12536 | FonNot2-3UTR-F | CGTCCGAGGGCAAAGGAATAGAGTAGTTATTACATGCCACTGACCC |
|  |  | FonNot2-3UTR-R | GCGACGCAGTATCCAACT |
| *Not2* | FOMG_12536 | FonNot2-JD-F | CGTAGCAGATGTTGGATTTGA |
|  |  | FonNot2-JD-R | AGGGCAATGGCACAGGTTC |
| *Not2* | FOMG_12536 | FonNot2c-F | ACTCACTATAGGGCGAATTGGGTACTCAAATTGGTTATTACCTGAAGATGAAAAGGAAGG |
|  |  | FonNot2c-R | CACCACCCCGGTGAACAGCTCCTCGCCCTTGCTCACAAGGATGCCGAGATGACGAT |
| *Not2* | FOMG_12536 | FonNot2c-JD-F | GACGGCAGGCTATGGTTTG |
|  |  | FonNot2c-JD-R | CTTCATGTGGTCGGGGTAG |
| *Not2* | FOMG_12536 | FonNot2-TZ-F | GAAGGACACGACGACCAC |
|  |  | FonNot2-TZ-R | AGGCAAGAAGGTAAGCAAGT |
| *Not2* | FOMG_12536 | FonNot2-RT-F | ACGAACCCTCTTGCTACGACAC |
|  |  | FonNot2-RT-R | GGCTGCGAAGGAATAACGTC |
| *Actin* | FOXG_01569 | FonActin-RT-F | GAGGGACCGCTCTCGTCGT |
|  |  | FonActin-RT-R | GGAGATCCAGACTGCCGCTCAG |
| *Opm12* | EU603504 | FonOpm12-RT-F | CGATTAGCGAAGACATTCACAAGACT |
|  |  | FonOpm12-RT-R | ACGGTCAAGAAGATGCAGGGTAAAGGT |
| *ClRps10* | HM036339 | ClRps10-RT-F | AGGCTCACCCTAAAAGAAGG |
|  |  | ClRps10-RT-R | GGTCAACACAAGGATCTTACT |
| *FMK1* | AF286533 | FonFMK1-RT-F | CGTGAGCGAGCAGTATGATAT |
|  |  | FonFMK1-RT-R | GATCTTCTTGATGGCGACCTT |
| *FGB1* | AB072452 | FonFGB1-RT-F | CAAGGCAACAGTGATGTCTCC |
|  |  | FonFGB1-RT-R | TGTGTCGGCGAGATCATCC |
| *FGA1* | AB072451 | FonFGA1-RT-F | CGCAACCGATACCACTCAGA |
|  |  | FonFGA1-RT-R | ACGCAGGTTCTCTTGGATGAT |
| *FOW2* | AB266616 | FonFOW2-RT-F | AAGAAGTCTGGCTCTAGTGGAA |
|  |  | FonFOW2-RT-R | GCAGAAGGCAAGGAATAAGGAA |
| *FVS1* | AB750777 | FonFVS1-RT-F | CCACGTCATCGGAAACAGAAA |
|  |  | FonFVS1-RT-R | CGCGAAGTGAATGACCAGAAC |
| *SHO1* | FOXG_06120 | FonSHO1-RT-F | CCTCGAAGTGTCGGATGTTAGC |
|  |  | FonSHO1-RT-R | GCATGGATCGTTGCTCATAACA |
| *MSB2* | FOXG_09254 | FonMSB2-RT-F | TCGTCCGTAATCTGACCTCCG |
|  |  | FonMSB2-RT-R | GTTGCCATCATCCGAGTTACCAT |
| *CHS1* | FOXG_19023 | FonCHS1-RT-F | TGATAACCTCGGATGGGGTG |
|  |  | FonCHS1-RT-R | CACAAGAAACCGGCAAAACG |
| *CHS2* | FOXG_04162 | FonCHS2 -RT-F | TTGGTTCATCCCCGATTTTG |
|  |  | FonCHS2-RT-R | GAGCATAGGGAATCCGACCA |
| *CHS4* | FOXG_05078 | FonCHS4-RT-F | TGCAACACCCACGATGTTTC |
|  |  | FonCHS4-RT-R | CAAGTTCTCGCTGGTATTGTGC |
| *CHS5* | FOXG_00113 | FonCHS5-RT-F | TCGTTGGTTTCCTGACTTTTGG |
|  |  | FonCHS5-RT-R | GACAAATCGTAAGCGACACCGT |
| *CHS6* | FOXG_04179 | FonCHS6-RT-F | CTTCCCCAAGCCCTGTTCA |
|  |  | FonCHS6-RT-R | TCGACGAGCAAACGACACG |
| *POD3* | FOXG_12260 | FonPOD3-RT-F | TTCTGTCCTTGTCTGTTGCGAG |
|  |  | FonPOD3-RT-R | GCCACGGGACACTTTCTCTC |
| *POD4* | FOXG_17180 | FonPOD4-RT-F | ATCACTTCTGCTGGCTTCTGGC |
|  |  | FonPOD4-RT-R | AGATGCCAAAGTCCTCGGGG |
| *POD5* | FOXG_17460 | FonPOD5-RT-F | TGTTGGCTTCTGGCCTCGTG |
|  |  | FonPOD5-RT-R | ACCTCCACCAGCCTGATTGCTA |
| *PODS1* | FOXG_10495 | FonPODS1-RT-F | GGCTCCAGACCGATGATAACTC |
|  |  | FonPODS1-RT-R | CACGCCTGGAGGGAATCTTG |
| *PODS2* | FOXG_13026 | FonPODS2-RT-F | CTATCGCTGCTTTCAACCTCAA |
|  |  | FonPODS2-RT-R | TCGTGGCGATGAATCAGAGG |
| *PODS3* | FOXG_13788 | FonPODS3-RT-F | CGAGGAGCAGAGCACTTACTTTG |
|  |  | FonPODS3-RT-R | TTATGAACACTGAGCAGGCATAGC |
| *FUB1* | FOXG_15248 | FonFUB1-RT-F | TGATTCTTCTTCGGGTGACGG |
|  |  | FonFUB1-RT-R | AGAGATAGGTAACTGCCCAGCTTG |
| *FUB4* | FOXG_15244 | FonFUB4-RT-F | CTGAGATGGCGAGGAAGATGA |
|  |  | FonFUB4-RT-R | AGTGAAGAGAAACAACCGTCTCAA |
| *FUB5* | FOXG_15243 | FonFUB5-RT-F | AAATGCCAAGTTGAGAGAGATCG |
|  |  | FonFUB5-RT-R | GCCGCTCGCTGCATAATATC |
| *FUB6* | FOXG_15241 | FonFUB6-RT-F | TTTCTGAGGGAAAGATCAAGTCG |
|  |  | FonFUB6-RT-R | GGGTCGCTAATCTTGAGCACC |
| *FUB8* | FOXG_15239 | FonFUB8-RT-F | CTCGCTCGCAATGAGACAGAA |
|  |  | FonFUB8-RT-R | CGAATGCCAGAACGGACTACA |
| *FUB10* | FOXG_21864 | FonFUB10-RT-F | GGTGAAGAGTCTGTGGTGTGTGAC |
|  |  | FonFUB10-RT-R | CAAGGCACGTCCAACAGGTC |
